# ANCA-Associated Intrahepatic Duct Injury Associated with Levamisole-Adulterated Cocaine

**DOI:** 10.1155/2020/8867183

**Published:** 2020-12-22

**Authors:** Joshua K. Salabei, Sara Khan, Afzal Khan, Zekarias T. Asnake, Troy J. Fishman, Jonathan D. Stromas, Zeeshan H. Ismail, John R. Leibach

**Affiliations:** ^1^University of Central Florida, School of Medicine, 6850 Lake Nona Blvd, Orlando, FL 32827, USA; ^2^North Florida Regional Medical Center, 6500 W Newberry Rd, Gainesville, FL 32605, USA

## Abstract

Damage to the liver or kidney can occur through direct toxic effects; however, damage can also be drug-induced immune-mediated. Levamisole-adulterated cocaine (LAC) is known to cause antineutrophil cytoplasmic antibody- (ANCA-) associated vasculitis and glomerulonephritis leading to acute kidney injury and end-stage renal disease. It remains unclear whether LAC is associated with hepatic duct damage. Here, we report a case with biopsy-proven evidence of intrahepatic duct damage months after being diagnosed with ANCA-associated crescentic and sclerosing glomerulonephritis caused by LAC use. This case represents the first report of LAC-induced ANCA-associated hepatic duct cholestasis in the setting of previous LAC-induced ANCA-positive glomerulonephritis.

## 1. Introduction

Among the many causes of hepatic duct damage leading to cholestasis, immune-mediated causes, particularly primary sclerosing cholangitis (PSC) and primary biliary cholangitis (PBC), have been well-characterized [1]. PSC is linked with autoantibodies such as antineutrophil cytoplasmic antibody (ANCA) which, in turn, is associated with other autoimmune diseases [2]. ANCA association has been described in variants of PSC, such as PSC high IgG4, PSC with features of autoimmune hepatitis, and small-duct PSC (SD-PSC) [3].

Direct damage to organ structures such as the hepatic duct or the glomerulus can occur with exposure to insults such as, but not limited to, antibiotics and herbals; however, damage could also be caused via a drug-induced immune-mediated fashion. For example, levamisole-adulterated cocaine (LAC) is known to cause perinuclear-ANCA- (p-ANCA-) associated vasculitis and glomerulonephritis leading to acute kidney injury and end-stage renal disease [4]. However, it remains unclear whether LAC is associated with hepatic duct damage and cholestasis. The following is a case presentation of a patient who presented with clinical signs and symptoms and biopsy-proven evidence of intrahepatic duct damage months after he was diagnosed with crescentic and sclerosing glomerulonephritis caused by LAC use.

## 2. Case Report

A 46-year-old male, who had just recently finished a 2-year prison term, initially presented at our hospital complaining of generalized weakness and fatigue. He also had blood-tinged sputum and cough with no fever. He reported using cocaine while incarcerated and had increased his consumption for the last 3 months leading to his presentation at our hospital. On presentation, his urine drug screen was positive for cocaine, and he was also found to have acute kidney injury with an elevated creatinine of 9.61, estimated glomerular filtration rate of 7 ml/min, and serum potassium of 7 meq/l. Chest CT scan showed cavitary lesions in the lungs, and serology testing was positive for antinuclear antigen (ANA) and ANCA and negative for antismooth muscle actin. He was positive for hepatitis C virus (HCV) antibody although HCV RNA was negative. He was started on dialysis, and, based on immunofluorescence stains on biopsied kidney that showed mostly fibrous crescents and a few cellular crescents, a diagnosis of microscopic polyangiitis secondary to LAC was made. The damage to his kidneys was irreversible, rendering him dialysis-dependent.

**Figure 1:**
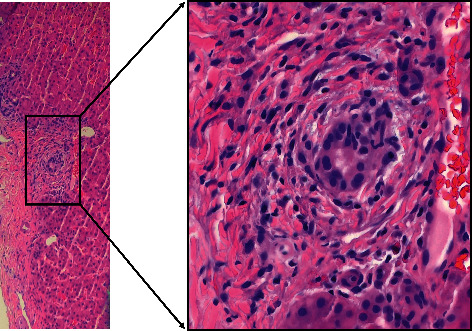
Representative stained image of biopsied liver. Characteristic onion skinning with focus of bile duct injury suggestive of ductular reaction to insult. Marked infiltration of neutrophils evident. Magnified image of boxed area is shown as image on the right. Blue staining indicates nuclei.

One month later, he presented again to our facility with generalized pruritus, dark urine, and several loose gray stools. On physical exam, his sclerae were icteric, and his skin appeared jaundiced but no skin lesions were noted. His pruritus had been resistant to multiple over the countermedications. Pertinent laboratory values at time of presentation and time of discharge (i.e., eight days later) are shown in Table 1. He was admitted and, per gastroenterology consult, started on treatment with ursodeoxycholic acid and cholestyramine. Based on his past serology, suspicion of a likely immune-mediated cause of his current presentation was high. Ultrasound of the abdomen, computed tomography (CT)/CT-angiography of the chest and abdomen, and magnetic resonance cholangiopancreatography (MRCP) did not show any obstruction in the form of bile stones or bile duct strictures. Follow-up liver biopsy for a confirmatory diagnosis showed isolated intrahepatic bile ducts with onion skinning with foci of bile duct injury with no significant fibrosis (Figure 1). Also, centrizonal canalicular cholestasis and mild periportal cholestatic ductular reaction were noted in the pathology report. The patient continued to respond to treatment with ursodeoxycholic acid and cholestyramine, and he was later discharged to follow-up with his gastroenterologist. Upon follow-up, his itching and jaundice had completely resolved.

## 3. Discussion

At the time of diagnosis with ANCA-positive glomerulonephritis, the patient was a heavy cocaine user. Serology and biopsy tests at that time confirmed the diagnosis of LAC-induced glomerulonephritis, similar to previously reported cases with similar timelines of LAC exposure [4]. Interestingly, his subsequent presentation with pruritis and jaundice coupled with his previous serology profile prompted a quick suspicion of an ANCA-associated liver pathology.

The characteristic onionskin thickening of the intrahepatic ducts seen on pathology is similar to that seen in PSC or SD-PSC [2]. Sole damage to and obstruction of the intrahepatic duct is suspected in the settings of normal ERCP and/or MRCP despite clinical manifestations and laboratory evidence pointing to cholestasis. A definitive diagnosis is usually made when examination of biopsied tissue shows damage to intrahepatic bile ducts (Figure 1). With the findings seen on pathology exam, coupled with the past serology report and clinical presentation, we were confident of an ANCA-associated intrahepatic duct damage.

This case demonstrates the challenges that sometimes must be overcome to make the diagnosis of cholestatic liver disease, especially when only intrahepatic ducts are involved. Imaging studies were negative in the presence of classical clinical presentation and laboratory evidence. Were it not for the past diagnosis of ANCA-associated glomerulonephritis, liver biopsy would have been waived, and only a focus on symptomatic treatment would have been pursued given that the patient's symptoms was improving and his laboratory values were trending to normality.

Some variants of classical PSC include PSC high IgG4 and PSC with features of autoimmune hepatitis characterized by high serum IgG4 and positive anti-*α*-SMA antibody, respectively [2]. In our case, anti-*α*-SMA antibody was negative (Table 1), and no evidence suggestive of elevated IgG was suspected. Interestingly, our patient had been infected with hepatitis C but had undergone treatment leading to undetected levels of hepatitis C viral RNA. Therefore, it is unlikely that his history of hepatitis C infection contributed to his current presentation because, in addition to his negative hepatitis C virus RNA, CT studies showed a normal-appearing liver. Also, no significant fibrosis was seen on biopsy, and prominent lymphoid aggregates typical of hepatitis C were not seen as well. In addition, changes well known to occur with cocaine use, for example, centrizonal necrosis, were not seen. These findings support our hypothesis that intrahepatic duct injury occurred via an ANCA-mediated fashion, rather than a direct toxic effect of cocaine or due to hepatitis C infection.

A rapid response to treatment with ursodeoxycholic acid and cholestyramine was observed in this case. Although we could not directly exclude the possibility of PSC, laboratory data obtained during a separate hospitalization for pneumonia eight months later showed complete normalization of the patient's laboratory values; total bilirubin 0.3, AST 24, ALT 19, and ALP 88, despite not on any immunosuppressive medication or extended treatment with ursodeoxycholic acid and cholestyramine. Such normalization is atypical of PSC which is usually a progressive disorder that ultimately leads to complications of cholestasis and hepatic failure [5–8]. Also, our patient has not had any symptoms concerning for ulcerative colitis known to be associated with up to 80% of individuals with PSC [5]. Still, there remains a possibility that his presentation is a very early presentation of classical PSC or a PSC variant or an entirely separate disease with a different course. That notwithstanding, we have reported a case of intrahepatic duct injury associated with LAC-induced glomerulonephritis. Very rarely has glomerulonephritis and hepatic duct pathology been reported in a single patient [9, 10], and no study, to the best of our knowledge, has shown the coexistence of intrahepatic duct injury in the setting of prior ANCA-positive LAC-induced glomerulonephritis.

## Figures and Tables

**Table 1:** Pertinent laboratory values.

Laboratory test	Values at presentation	Values at discharge	Reference values
Total bilirubin	13.0	2.6	(0.2–1.0 mg/dL)
Direct bilirubin	11.1		(0.0–0.2 mg/dL
Alkaline phosphatase	520	233	(45–117 units/L)
AST	196	196	(15–37 units/L)
ALT	235	235	(16–61 units/L)
PT	10.3		(9.0–12.5)
INR	1.0		(1.0–1.5)
Anti-MPO (p-ANCA)^#^	>100		(0.0–9.0)
p-ANCA^¶^	1 : 80		(<1 : 20)
Antiproteinase 3 (c-ANCA)^#^	8.7		(0.0–3.5)
c-ANCA	<1 : 20		(<1 : 20)
Anti-*α*-SMA	14		(0–19)
Anticentromere	7.3		(0.0–0.9)

AST: aspartate aminotransferase; ALT: alanine aminotransferase; PT: prothrombin time; INR: international normalized ratio; MPO: myeloperoxidase; ANCA: antineutrophil cytoplasmic antibodies; SMA: smooth muscle actin. ^#^By enzyme immunoassay. ^¶^By indirect immunofluorescence assay.
